# Skeletal Muscle Metastasis From Renal Cell Carcinoma: A Case Series and Literature Review

**DOI:** 10.3389/fsurg.2022.762540

**Published:** 2022-03-03

**Authors:** Juan Sun, Zimu Zhang, Yu Xiao, Hanzhong Li, Zhigang Ji, Penghu Lian, Xuebin Zhang

**Affiliations:** ^1^Division of General Surgery, Department of Surgery, Peking Union Medical College Hospital, Peking Union Medical College and Chinese Academy of Medical Sciences, Beijing, China; ^2^Department of Pathology, Peking Union Medical College Hospital, Peking Union Medical College and Chinese Academy of Medical Sciences, Beijing, China; ^3^Division of Urology, Department of Surgery, Peking Union Medical College Hospital, Peking Union Medical College and Chinese Academy of Medical Sciences, Beijing, China

**Keywords:** renal cell carcinoma, skeletal muscle metastasis, biopsy, lesion excision, adjuvant treatment

## Abstract

**Objectives:**

Skeletal muscle metastasis (SMM) from renal cell carcinoma (RCC) has been rarely reported. This case series was performed to increase the clinicians' understanding of its clinical features and treatments.

**Methods:**

We evaluated the clinical presentations, diagnoses, and treatments of 2 patients with SMM from RCC in our hospital and 39 cases reported in the literature.

**Results:**

Among the 41 patients, 4 (9.76%) were women and 37 (90.24%) were all men. The average age was 60.5 ± 12.6 years old (range from 7 to 81). The size of tumors varied from 1 to 28 cm, and the metastatic sites of 6 (14.63%) cases were in the heads, 20 (48.78%) in the limbs, 9 (21.95%) in the trunks, 3 (7.32%) in the buttock, and the other 3 (7.32%) were multiple sites. The mean of intervals between the RCC and the discovery of the first SMM was 73.61 months. More than half of the patients (25, 60.98%) were diagnosed by MRI and 25 (60.98%) patients performed a biopsy of the mass to establish the diagnosis. Finally, 30 (73.17%) cases performed mass excision. Then the adjuvant therapy was performed in 18 patients including immunotherapy, radiotherapy, chemotherapy, and targeted therapy. The median follow-up after SMM was 9 months (P25, P75: 5, 23), in which the longest survival time of patients with SMM of RCC was 8 years while the shortest was only 3 months.

**Conclusion:**

The characteristic clinical feature of SMM from RCC is asymptomatic masses or swelling with a long history which can be preoperative suspiciously diagnosed by MRI. The rapid biopsy of suspected lesions, determination of other metastasis sites, resection of metastasis, and systematic treatment are the recommended treatments of it.

## Introduction

Kidney cancer is one of the most common malignant tumors in the urology system, accounting for ~3% of all adult malignant tumors worldwide (1), and renal cell carcinoma (RCC) accounts for approximately 80% of all kidney cancers (2), with laparoscopic resection as the main treatment, followed by immunotherapy or targeted therapy (3–5). However, tumor metastasis is still a conundrum, as it determines the treatment strategy and overall prognosis on patients with RCC (6). As it was reported in 2020, the 5-year survival rate after surgical treatment for the localized disease was 92%, while that with metastasis was only 12% (7). The common metastatic sites of RCC are the lungs, bones, lymph nodes, liver, adrenal glands, and brain, but rarely to the skeletal muscles (8).

We herein describe two cases of skeletal muscle metastasis (SMM) from RCC from 1983 to 2020 in the Peking Union Medical College Hospital and further review the literature regarding SMM from RCC to discuss the clinical manifestations, diagnosis, and treatments of this condition. Because case reports can be an essential source of information for the optimum care of patients for rare events, we aim to increase the clinicians' understanding of the optimal methods of diagnosis and treatment in terms of SMM from RCC.

## Materials and Methods

### Patients

Two patients were diagnosed with SMM from RCC between January 1983 and December 2020 at Peking Union Medical College Hospital. We also identified 39 cases with sufficient medical history information reported in the English language literature from 1979 to 2020; these case reports were retrieved from PubMed and GeenMedical.

### Methods

Age and sex of the 41 patients; site and size of SMM; symptoms and intervals; diagnostic methods; whether a biopsy was done; therapies, postoperative treatments, and follow-up were documented and retrospectively analyzed. The medical services performed for the two patients treated in our hospital were recorded in detail.

## Results

### Case 1

**Figure 1:**
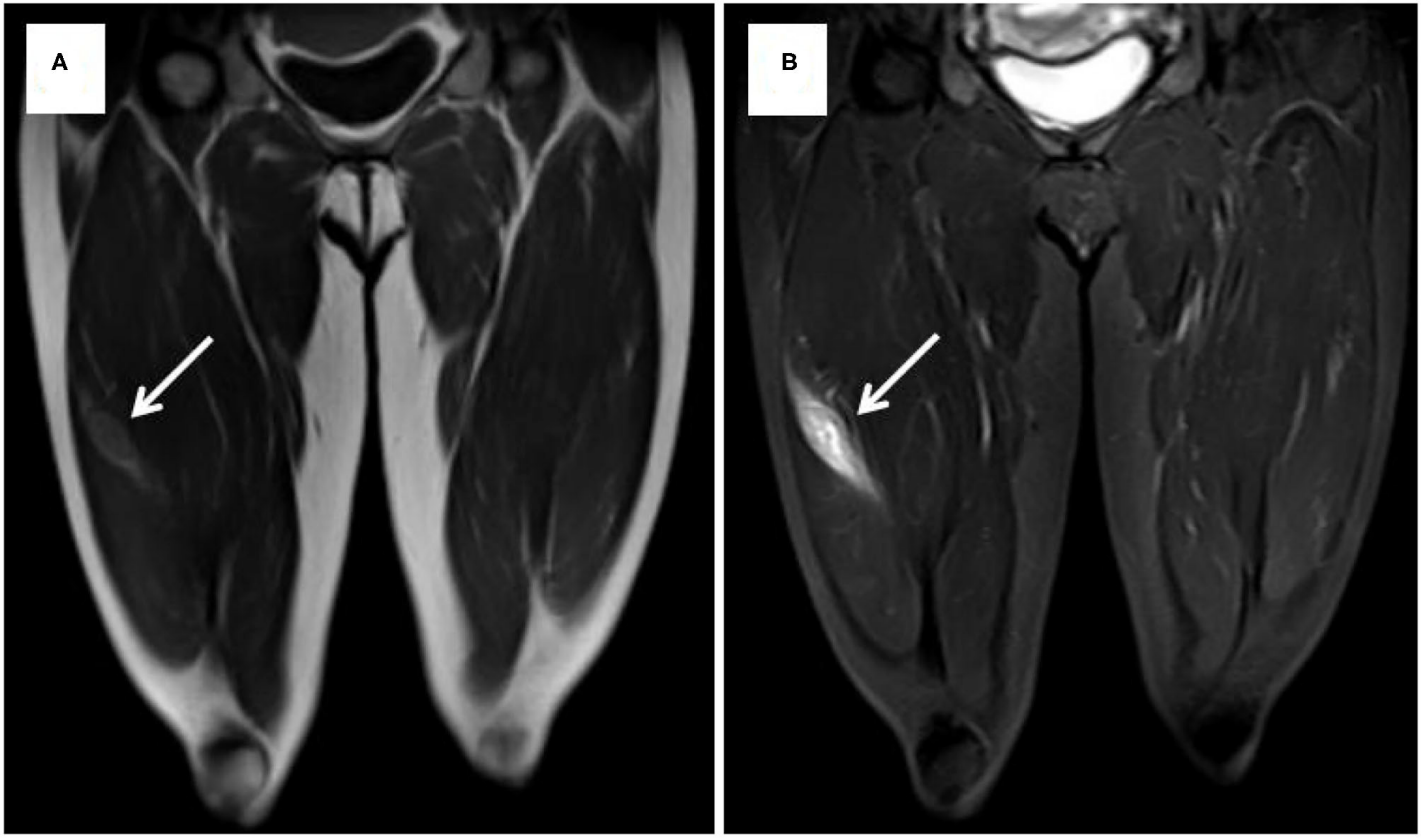
MRI showed slightly high signal intensity on T1-weight image **(A)** and high signal intensity on T2-weight image **(B)**.

**Figure 2:**
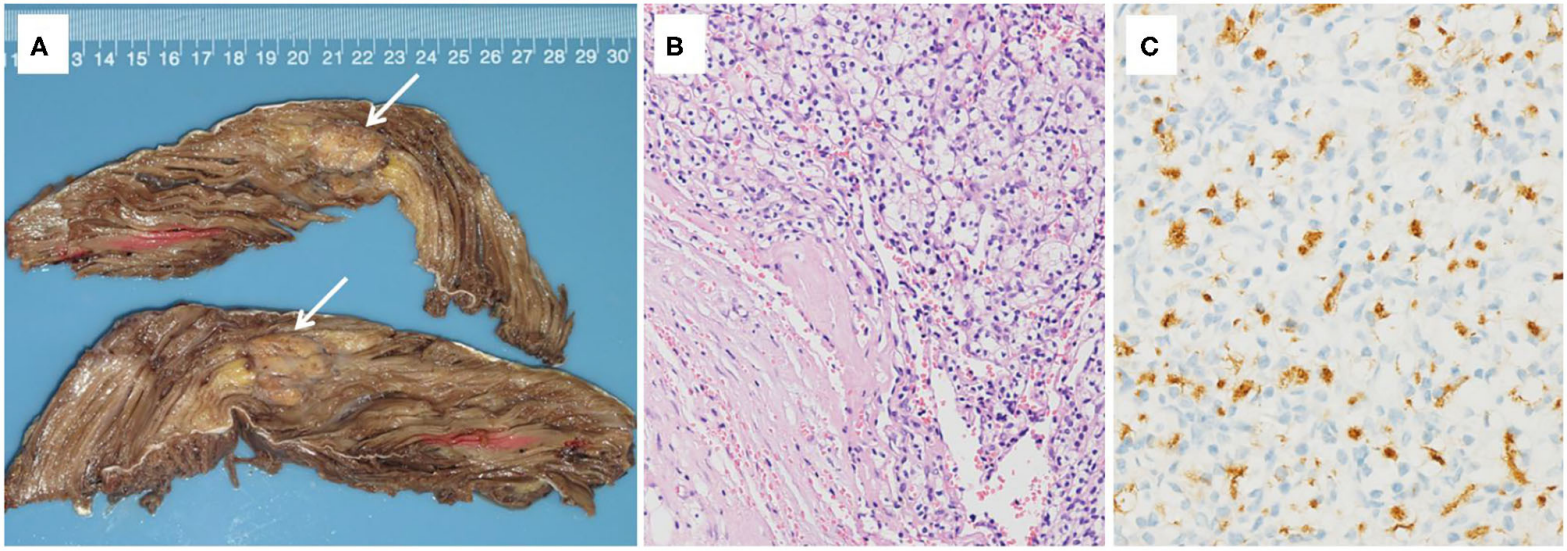
**(A)** Gross pathology of the right vastus lateralis muscle, revealing a mass measured 2.6 × 2.3 × 2.1 cm in size with a grayish-yellow color, separated by fibrotic scar. **(B)** Histopathologic appearance of the renal cell carcinoma (RCC) metastasis in the right vastus lateralis muscle (hematoxylin-eosin [H&E] staining, original magnification X100). **(C)** Histochemical assay result of the mass was positive for RCC (RCC X200).

Case 1 was a 44-year-old Chinese woman who was presented to our hospital with a painless mass on her right leg in September 2011. On physical examination, the mass was about 4 cm in diameter with minor mobility. The patient underwent left radical nephrectomy due to renal clear cell carcinoma (T1N0M0) in 2003. Ultrasonography of the right leg showed a hypoechoic mass measuring 3.0 × 1.8 × 2.1 cm in the skeletal muscle. Further MRI revealed a heterogeneous signal mass, 4.0 × 2.5 cm in size, which is located in the vastus lateralis muscle and showed as slightly high-signal intensity on T1-weight image and high-signal intensity on T2 (Figure 1). Chest and abdominal computed tomography scans revealed no evidence of metastasis. A neoplasm resection was performed and a pathologic examination of the mass from the right leg demonstrated clear cell type RCC (Figure 2). Immunohistochemical results showed the following: CD10(+), EMA(+), RCC(+), Vimentin(+), Ki-67 index (10%), and AE1/AE3 (focal +). Sorafenib was given 400 mg bid for 2.5 months but was ceased due to adverse effects of pharyngalgia, hair loss, and finger desquamation. During the follow-up, the metastases were found in her left thyroid (2012), pancreas (2012), right kidney (2013), dorsal muscle (2013), the same position of the right leg (2014), liver (2017), lung (2018), and brain (2018). As for the treatment for these metastatic sites, left thyroidectomy was done in 2012, gamma knife (a kind of stereotactic radiosurgery) for the pancreas, radiofrequency ablation for the right kidney, surgical resection for SMM of the dorsal muscle and leg, TACE (transarterial chemoembolization) for the metastatic lesions of the liver and axitinib was taken for 3 months, radiotherapy for pulmonary metastasis in 2018, and finally in her last time of the life, she chose traditional Chinese medical herbal treatment for the intolerant symptoms. The International Metastatic RCC Database Consortium (IMDC) class of this patient was intermediate and she died in 2019, after 8 years of our first mass excision surgery.

### Case 2

**Figure 3:**
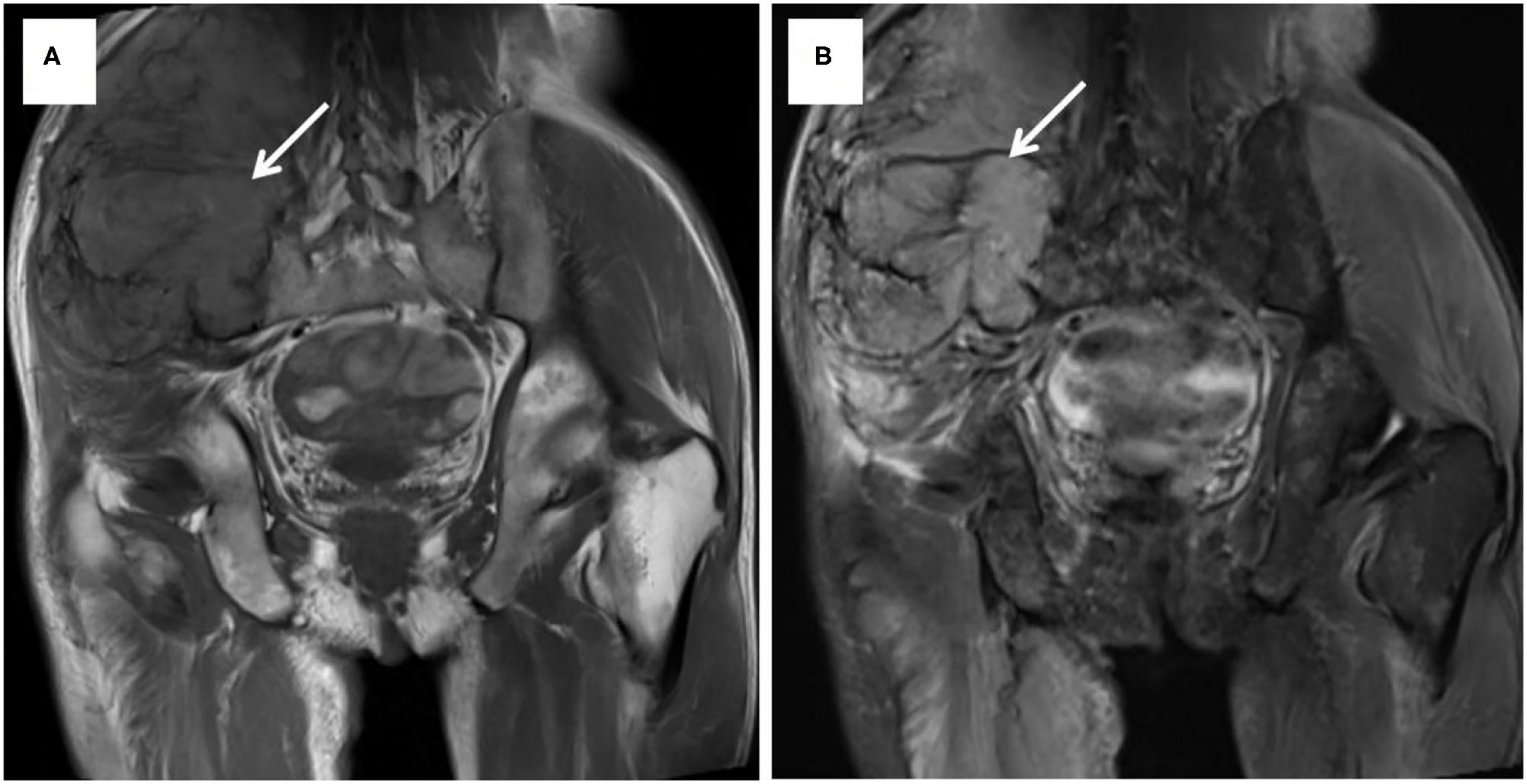
MRI showed a mixed signal from the right psoas to gluteus maximus, involving the right iliac bone. T1-weight image **(A)** and T2-weight image **(B)**.

Case 2 was a 63-year-old man who was admitted to our hospital on July 15, 2014, with a 9-month history of a mass in the right iliac accompanied by local swelling and raised skin temperature. Physical examination on arrival at our hospital revealed a mass about 13 × 11 cm in size in the lateral area of the right iliac pterion, which was soft, inactive, and painless. The US revealed that low echo and abundant blood flow signals can be seen in the subcutaneous tissue layer of the right hip. MRI showed that an oval mixed signal about 13.3 × 11.1 × 14.6 cm could be seen from the right psoas to gluteus maximus, involving the right iliac bone (Figure 3). The PET-CT of the whole body showed that there was osteolytic bone destruction of the right iliac bone which was considered to be bone metastases; a round mass behind the middle and upper left kidney which was suspected to be RCC and no other hypermetabolic lesion in the whole body. Ultrasound-guided biopsy of mass suggested a small number of atypical tumor cells, but the source was uncertain. Because of the anemia of this patient (hemoglobin: 52 g/L, normal: 120–150 g/L) and huge size of the tumor, the operation was very difficult and with high risk. Finally, the patient and their families chose to perform only tumor biopsy to confirm the pathology. Therefore, the patient underwent a right iliac tumor biopsy under local anesthesia and the specimens confirmed that the tumor was from RCC. Then the patient was discharged and chose no further more treatment. The IMDC class of this patient was intermediate and he was died 3 months after the discharge.

### Previously Published Cases

We identified 39 cases of SMM from RCC reported in English language publications; including our two cases (40 and 41), there were 41 cases (Table 1). In these 41 cases, 4 (9.76%) were females and the other 37 (90.24%) were all males. The average age was 60.5 ± 12.6 years old (range from 7 to 81) when SMM was diagnosed, and detailly 6 (14.63%) were <50 years old and 35 (85.37%) were ≥50 years old. The size of tumors varied from 1 to 28 cm. As for the location of the SMM, 6 (14.63%) were in the heads, 20 (48.78%) in the limbs, 9 (21.95%) in the trunks, 3 (7.32%) in the buttock, and the other 3 (7.32%) were multiple sites. Concerning the intervals between the RCC and the discovery of the first SMM, the mean interval was 73.61 months. Nine patients were presented with SMM at the beginning and the longest one was 21 years. More than half of patients (25, 60.98%) were diagnosed by MRI and 25 (60.98%) patients performed biopsy of the mass to establish the diagnosis. In the treatments of these 41 patients, 30 (73.17%) performed mass excision, 5 did not receive any surgical treatments, 2 underwent nephrectomy alone, and the operative information of the remaining 4 patients was not available. Followed by the postoperative treatment, adjuvant therapy was performed in 18 patients including immunotherapy, radiotherapy, chemotherapy, and targeted therapy. Moreover, the data of 19 patients in follow-up was not available, but the median follow-up after SMM was 9 months (P25, P75: 5, 23) in the last 22 patients in which the longest survival time of patients with SMM of RCC was 8 years while the shortest was only 3 months.

**Table 1:** Reported cases of skeletal muscle metastasis (SMM) from renal cell carcinoma (RCC) from literatures.

	**Age/Sex**	**Site**	**Size (cm)**	Symptoms and Intervals^*^	**Diagnosis**	**Biopsy**	**Therapy**	**PO treatment**	**Other organ metastases (Y/N)**	**Follow-up**
1	58/M	Center forearm (brachioradialis)	4 × 2× 1.7	A slow growing mass, 8 years	US, MRI	No	Lesion excision	IT	Y	Died 15 months PO (9)
2	74/M	Right back (erector spinae)	5.6 × 4.3 × 2.4	Painless swelling, 28 months	US, CT, MRI	Yes	Wide resection	NA	N	NA (10)
3	74/M	Center leg (soleus muscle)	10.6 × 3.9 × 5.7	A growing mass, 3 years	US, MRI	Yes	Wide resection	None	N	NA (11)
4	67/M	Right forearm (digitorum muscle)	6.6 × 2.4 × 1.9	Pain, 4 years	X-ray, MRI	Yes	Lesion excision	NA	Y	NED after 8 months (12)
5	48/M	Multiple sites	NA	Pain and limping, 4 years	X-ray, MRI	Yes	NA	NA	N	NA (13)
6	67/M	Right thigh (gluteus maximus)	16.5x7.5x6.7	A painful hard mass, 9 years	MRI	Yes	Total excision	RT, IT, TT	N	NA (14)
7	74/M	Right gluteus medius	10 × 8.5 × 5.5	Painless swelling, 7 months	MRI	Yes	Total excision	IT (IFN-a)	N	NA (14)
8	7/M	Center deltoid muscle	3 × 2 × 2	Flank pain, together	MRI	No	NR and excision	IT (IFN-a), TT	Y	Died 9 months PO (15)
9	76/F	Right thigh	4.5 in diameter	Indolent swelling, 1year	US, CT	Yes	No surgery	TT	Y	NA (16)
10	58/M	Center biceps femoris muscle	28 × 17 × 7	A growing mass, 9 years	PET/CT, MRI	Yes	Surgical resection	NA	Y	NA (17)
11	63/M	Center psoas muscle	7	A growing nodule, 11 years	CT	No	Surgical resection	NA	Y	NED after 18 months (18)
12	71/M	Right facial muscle	4.1	A palpable mass, 12 years	PET/CT, MRI	Yes	NA	NA	N	NA (19)
13	58/M	Center adductor muscle	5	A painless mass, 5 years	PET/CT, MRI	No	Surgical resection	NA	Y	NA (20)
14	58/M	Posterior aspect of his center leg	2 × 4	A painful swelling, together	MRI	Yes	NR and excision	RT, IT	N	NED after 1 year (21)
15	60/M	Right infraspinatus muscle	NA	Weight loss, anorexia, together	PET/CT	No	Right nephrectomy	IT, ChT	Y	Progressed 5 months PO (22)
16	71/M	Center posterior thigh	16 × 7.5	A mass with pain, 21 years	MRI	Yes	NA	NA	N	NA (23)
17	65/M	Right gluteus maximus	3.8 in diameter	A growing mass, 1 year	CT, MRI	No	Wide resection	NA	Y	NA (24)
18	63/M	Multiple sites	NA	Lower-back pain, 19 years	X-ray, CT	Yes	NA	NA	Y	NA (25)
19	53/M	Right longissimus thoracis	15	Fatigue and anemia, together	CT, MRI	Yes	Nephrectomy	IT (IFN-a)	Y	Died 20 weeks post operatively (26)
20	73/F	Right leg (quadriceps muscle)	5 × 4 × 9	A painless mass, 6 years	CT, MRI	Yes	Surgical resection	IT	N	NED after 6 months (27)
21	50/M	Center thigh (vastus medialis)	4.0 × 3.8	A painless mass, 14 years	US, MRI	No	Wide resection	None	Y	NED after 2 months (28)
22	63/M	Right psoas muscle	1.5	No symptoms, 14 years	CT, MRI	No	Surgical resection	NA	N	NA (29)
23	69/M	Center shoulder (trapezius muscle)	6 × 3 × 3	Swelling, 2 years	US, CT, MRI	Yes	No surgery	RT, IT (IFN-a)	Y	NA (30)
24	60/M	Center face (masseter muscle)	1.5 in diameter	A painless mass, 6 months	CT	No	Local exicision	IT (IFN-β, IL-2)	N	NA (31)
25	81/M	Center arm (triceps muscle and the brachioradial muscle)	3 × 4 cm 1 × 1 cm	A painless mass, 15 years	CT, MRI	Yes	Wide resection	NA	Y	NED after 1 year (32)
26	49/M	Center face (masseter muscle)	4 × 4	Swelling, together	CT, MRI	Yes	Lesion excision	None	Y	Died 3 years PO (33)
27	57/M	Center face (masseter muscle)	1.0	No symptoms, 4 years	US, CT	No	Lesion excision	IT (IFN-a)	Y	NA (34)
28	58/M	Center psoas muscle	5.0	Dull pain, 14 years	US, CT	Yes	Surgical resection	NA	N	NA (35)
29	55/M	Center buttock (gluteus maximus)	4.7 in diameter	A painless mass, 12 years	US	Yes	Surgical resection	None	N	NED after 4 months (36)
30	41/M	Tongue muscle	2 in diameter	Hemosputum, 3 years	laryngoscopy	Yes	No surgery	IT (IFN-a)	Y	Died 6 months PO (37)
31	57/M	Right shoulder (Trapezius)	4 × 3	A painful mass, 10 months	CT, MRI	No	Wide resection	NA	N	NA (38)
32	63/F	Multiple sites	NA	A painless mass, 16 years	CT	No	Wide resection	NA	N	Alive 6 years post operatively (39)
33	63/M	Extraocular muscles	Focal nodular enlargement	Diplopia, together	US, CT, MRI	Yes	No surgery	RT, ChT	Y	Died 4 months after initial presentation (40)
34	69/M	Center thigh muscles	NA	A solitary hard mass, 12 months	CT	No	Surgical resection	IT (IFN-a)	Y	Recurrence 6 months PO (41)
35	55/M	Center thigh muscles	NA	NA, 33 months	NA	NA	Surgical resection	NA	N	NED after 93 months (42)
36	46/M	Center thigh muscles	NA	NA, 196 months	NA	NA	Lesion excision	NA	N	NED after 45 months (42)
37	74/M	Center arm (triceps muscle)	6 × 5 × 6.5	A growing mass, together	X-ray, CT	Yes	NR and excision	NA	Y	NA (43)
38	63/M	Right thigh muscles	NA	Pain and swelling, 69 months	CT	Yes	Wide resection	RT, ChT	N	NED after 12 months (44)
39	62/M	Right arm (biceps muscle)	10 × 8	A painful mass, together	Arteriogram	Yes	NR and excision	RT, ChT	Y	Recurrence 9 months PO (45)
40	44/F	Right leg (vastus lateralis muscle)	4.0 × 2.5	A painless mass, 8 years	US, MRI	No	Mass excision	TT, ChT	Y	Died 8 years after the first surgery
41	63/M	Right iliac (psoas to gluteus)	13.3 × 11.1 × 14.6	A mass with swelling, together	US, MRI	Yes	No surgery	No	N	Died 3 months after the biopsy

* *Interval between the primary tumor and the discovery of the first skeletal muscle metastasis; PO, Postoperative; NR, Nephrectomy; NED, no evidence of disease; NA, information not available; RT, Radiotherapy; IT, Immunotherapy; ChT, chemotherapy; TT, targeted therapy*.

## Discussion

The SMM from RCC occurs in <1% of patients and is described only in case reports (9). The reported reasons for the rarity of SMM from RCC can be summarized as the high vascularization of muscles; the lactic acid production in skeletal muscle may lead to angiogenesis resistance (46, 47); high concentrations of free radicals, local temperature fluctuations, skeletal muscle-derived peptide factor, protease inhibitors, lymphocytes, and natural killers may inhibit metastasis (12, 48–50); specific receptors which affect the metastasis potential of RCC may be missing or scarce in muscles (51, 52). Additionally, a study also suggested that the damage of skeletal muscle may increase the risk of metastasis in this location (53).

Patients with SMM from RRC usually have no symptoms in the early stage, so the metastases are usually found only when they turn to larger sizes and cause symptoms, such as local pain, swelling, or obvious mass. There is no consensus on the common site of metastasis, but according to our study, limbs seem to be its favorite site (48.78%), which differed from the trunk muscles (83.3%) of the study group in Haygood's report (54). In our review, 22 cases had other organ metastases before or after the discovery of SMM from RCC. However, in Haygood's report, only one in 21 of their own series had skeletal muscle–only metastasis, while more than half of the patients in his review group had metastases in other organs.

Because of the rarity and unpredictability, diagnosis of malignant SMM is rather challenging. MRI is an important imaging modality to distinguish primary soft tissue tumors from metastatic tumors. Surov et al. (55) studied the imaging manifestations of muscle metastasis in 461 cases of different primary tumors, which included 38 cases of metastatic RCC, and showed that 48.3% of the muscle metastasis was homogeneously isointense when compared with unaffected muscle on T1-weight images of MRI, but on the T2-weighted, 81.6% of the SMM showed high signal intensity, while PET/CT has a unique value in evaluating distant metastasis. Aurangabadkar and Ali. (56) reported an unusual case of extensive skeletal muscle metastases demonstrating as a focal hypermetabolic lesion.

Positron emission tomography/computed tomography (PET/CT) and MRI can help understand the morphology of tumors, but the pathology remains the gold standard for diagnosis. Therefore, for SMM suspected from RCC, the biopsy is still necessary to diagnose and differentiate RCC metastasis from other soft tissue tumors (10). Biopsy provides the most direct evidence of the nature of lesions compared to non-invasive imaging modalities. However, if the needle biopsy confirms malignancy, the puncture tract should also be excised in case of the seeding of tumors (17).

Renal cell carcinoma is aggressive and almost 25% of patients are diagnosed with distant metastasis (57). The median survival time of untreated patients with metastatic RCC was 6–12 months, and the 5-year survival rate was <20% (58). But for local metastases, surgical resection is beneficial to prolong survival (9) and five-year survival rates are between 35 and 50% after surgical therapy for solitary metastasis (17, 59). Besides, it was also reported that in patients with multiple and non-lung-only metastasis, complete metastasectomy can bring the benefit of a 5-year survival rate to 32.5 vs. 12.4% without complete resection (60). Among the 41 reported cases in our study, 30 (73.17%) cases performed surgical resection of the SMM, and the mean overall survival time of five untreated patients was just 4.6 months, while in patients with surgical resection of SMM it was 27 months.

Apart from surgical resection, systemic treatment with targeted therapy, immunotherapy, chemotherapy, or radiotherapy might be applied in patients with metastatic RCC, since it provides potential benefit for long survival (61). RCC itself is not sensitive to radiotherapy and chemotherapy and targeted therapy is the mainstream treatment for metastatic RCC. However, the complete remission rate of single targeted therapy is only 1–3%, which can only increase to 2–6% even if combined with immunotherapy (62). Therefore, for those residual or unresectable metastatic lesions which are essential for overall survival, intensity-modulated radiotherapy (IMRT) was proved to be able to achieve great local control, especially the image-guided radiotherapy (IGRT) for RCC bone metastasis (63). IGRT is an on-line precise radiotherapy technology for tracking the target area, which can ensure the reduction of the external safety boundary of the spinal cord without affecting the reliability of the radiotherapy accuracy and allows the generation of highly conformal dose distribution for concave target volumes wrapped around organs at risk (64). All in all, the unpredictable behavior of RCC suggests that patients need to be thoroughly followed up because early recognition of tumor recurrence will be more effective for surgical resection and systemic treatment (16).

## Conclusion

This study indicated that the clinical manifestation of SMM from RCC generally involves a long history of asymptomatic masses or swelling; MRI may be more effective and recommended for its preoperative diagnosis. The recommended managements include rapid biopsy of the suspected lesions, identification of sites of other metastasis, resection of the metastatic masses, and systemic treatment.

## Data Availability Statement

The original contributions presented in the study are included in the article/supplementary material, further inquiries can be directed to the corresponding author.

## Author Contributions

JS and ZZ were responsible for collecting, sorting out data, and writing the article. YX and HL were responsible for collecting data and pathologic pictures. PL and XZ were responsible for putting forward ideas and reviewing articles and were the co-corresponding authors of this article. All authors contributed to the article and approved the submitted version.

## Funding

This work was funded by Beijing Municipal Science and Technology Commission: Research and development of transurethral endoscopic surgical robot prototype No: Z191100007619044.

## Conflict of Interest

The authors declare that the research was conducted in the absence of any commercial or financial relationships that could be construed as a potential conflict of interest.

## Publisher's Note

All claims expressed in this article are solely those of the authors and do not necessarily represent those of their affiliated organizations, or those of the publisher, the editors and the reviewers. Any product that may be evaluated in this article, or claim that may be made by its manufacturer, is not guaranteed or endorsed by the publisher.
